# Targeting SLC4A4: A Novel Approach in Colorectal Cancer Drug Repurposing

**DOI:** 10.3390/cimb47010067

**Published:** 2025-01-20

**Authors:** Krunal Pawar, Pramodkumar P. Gupta, Pooran Singh Solanki, Ravi Ranjan Kumar Niraj, Shanker L. Kothari

**Affiliations:** 1 Amity Institute of Biotechnology, Amity University Rajasthan, SP-1, Kant Kalwar, RIICO Industrial Area, NH-11C, Jaipur 303002, Rajasthan, India; krunal.24.6@gmail.com (K.P.); rrkniraj@gmail.com (R.R.K.N.); 2 School of Biotechnology and Bioinformatics, D Y Patil Deemed to be University, Plot 50, Sector 15, CBD Belapur, Navi Mumbai 400614, Maharashtra, India; 3 Bioinformatics Center, Birla Institute of Scientific Research, Jaipur 302001, Rajasthan, India; pooransingh@bisr.res.in; 4 Department of Bioengineering and Biotechnology, Birla Institute of Technology, Mesra, Off Campus Jaipur, Jaipur 302001, Rajasthan, India

**Keywords:** colorectal cancer, SLC4A4, drug design, molecular docking, molecular modeling

## Abstract

Background: Colorectal cancer (CRC) is a complex and increasingly prevalent malignancy with significant challenges in its treatment and prognosis. This study aims to explore the role of the SLC4A4 transporter as a biomarker in CRC progression and its potential as a therapeutic target, particularly in relation to tumor acidity and immune response. Methods: The study utilized computational approaches, including receptor-based virtual screening and high-throughput docking, to identify potential SLC4A4 inhibitors. A model of the human SLC4A4 structure was generated based on CryoEM data (PDB ID 6CAA), and drug candidates from the DrugBank database were evaluated using two computational tools (DrugRep and CB-DOCK2). Results: The study identified the compound (5R)-N-[(1r)-3-(4-hydroxyphenyl)butanoyl]-2-decanamide (DB07991) as the best ligand, demonstrating favorable binding affinity and stability. Molecular dynamics simulations revealed strong protein–ligand interactions with consistent RMSD (~0.25 nm), RMSF (~0.5 nm), compact Rg (4.0–3.9 nm), and stable SASA profiles, indicating that the SLC4A4 structure remains stable upon ligand binding. Conclusions: The findings suggest that DB07991 is a promising drug candidate for further investigation as a therapeutic agent against CRC, particularly for targeting SLC4A4. This study highlights the potential of computational drug repositioning in identifying effective treatments for colorectal cancer.

## 1. Introduction

Colorectal cancer (CRC) is one of the most commonly diagnosed malignant cancers worldwide, and is a genetically, anatomically, and transcriptionally heterogeneous disease, with increasing incidence and mortality [1,2]. By 2030, the burden of CRC is predicted to increase by 60% [3]. Depending on the stage at which the tumor was diagnosed, a CRC patient’s prognosis varies greatly. Researchers have been able to develop specific drugs and therapeutic regimens and prove that they are effective in treating CRC, as research into the disease continues to progress [4].

Early detection and prevention are paramount to reducing CRC mortality; this has led to the development and refinement of various screening methods, including for stool- and blood-based biomarkers [5]. The therapeutic landscape for CRC has evolved significantly over the years. Standard treatment protocols typically involve a combination of surgery, chemotherapy, radiation therapy, and targeted therapies, with emerging non-invasive approaches like Focused Ultrasound showing promise as potential additions to current modalities [6]. Surgery remains the cornerstone for localized colon cancer, often followed by adjuvant chemotherapy for stage II and III cancers to reduce recurrence risk [7]. Chemotherapeutic agents such as 5-fluorouracil (5-FU), oxaliplatin, and irinotecan are commonly used, either alone or in combination (e.g., FOLFOX, FOLFIRI) [8,9]. Targeted therapies, including monoclonal antibodies against EGFR (cetuximab, panitumumab) and VEGF (bevacizumab), have shown efficacy in metastatic CRC [10]. Additionally, immunotherapies like checkpoint inhibitors (e.g., pembrolizumab) are being explored, especially in mismatch repair-deficient (dMMR) and microsatellite instability-high (MSI-H) tumors [11]. Despite these advancements, resistance to current treatments and adverse side effects necessitate the continuous evaluation of new therapeutic compounds. Research efforts are increasingly becoming focused on discovering novel agents that can enhance treatment efficacy, reduce toxicity, and overcome resistance mechanisms [12].

Recent studies in the field of molecular pathology have introduced thousands of tumor biomarkers associated with the progression or prognosis of different types of cancers [13]. Gene chip analysis is a gene-detection technique that has been in use for >10 years; this technique can detect the expression information all the genes within the same sample time-point, and is particularly suitable for screening differentially expressed genes [14]. The Gene Expression Omnibus (GEOs) is a repository of gene expression data, wherein a large number of microarray data have been deposited and stored [15]. A considerable amount of microarray data on CRC have been accumulated, and SLC4A4 has been identified as a reliable biomarker for CRC. A noteworthy association has been observed between SLC4A4 expression and reduced survival in patients with colorectal cancer (CRC), as well as worse prognosis in patients with gastric, ovarian, lung, and breast cancer. SLC4A4 may be involved in tumor suppression and prognostic prediction for several cancers, including CRC [16,17]. Low SLC4A4 expression, which correlates with increased lymph node and distant metastasis, and regulates partial epithelial–mesenchymal transition (EMT) phenotypes that are critical for cancer cell migration and invasion, suggests that SLC4A4 functions as a tumor suppressor gene and serves as a potential prognostic biomarker [18].

Several studies have demonstrated that the dysregulation of bicarbonate transporters can be used in cancer treatment as both a diagnostic and a therapeutic mechanism. An analysis of public expression data indicates that SLC4- and SLC26-family transporters are widely expressed in breast, lung, and colon cancer patients [19,20]. Sodium-driven, acid-extruding bicarbonate transporters like SLC4A4 prevent intracellular acidosis (low pH in cells) [21]. Tumors typically exhibit a decrease in interstitial pH in comparison to healthy tissues. The lowest pH that tumor tissue can contain is 5.6. Tumor acidity is emerging as a key driver of cancer progression because it can favor the selection of malignant cancer cells and, at the same time, can affect the composition and function of stromal cells present in the tumor microenvironment (TME) [22,23]. In innate and adaptive immune cells infiltrating tumors, acidity may blunt their antitumor response, resulting in immune escape [24,25,26]. T-cells and natural killer cells become dysfunctional in low-pH environments or when exposed to high levels of lactate, favoring the expansion of myeloid cells, which have immunosuppressive properties [27,28,29,30]. As tumor acidity can significantly impact the effectiveness of immune checkpoint inhibitors, targeting the main pH modulators in immunotherapy and antitumor immunity is of fundamental importance [31].

Due to the promise of reduced costs and expedited approval schedules, drug repositioning is a rapidly growing field, as it involves discovering, validating, and marketing previously approved drugs for new indications [32]. The advent of computational methodologies in drug design, discovery, and development has provided scientists with a streamlined approach to initiate drug repurposing efforts. This rationalization of the process has notably reduced the probability of failed attempts in repurposing drugs [33]. Computational techniques like molecular docking, dynamics simulations, in silico ADMET, and drug-likeness predictions are predominantly employed in the discovery and development of drug candidates sourced from various databases [34]. Given the abundance of computer-aided drug design tools and combinatorial chemistry techniques, there is a diverse range of therapeutic possibilities within reach. Our study delves into the realm of SLC4A4 inhibitors through computational methods, assessing their pharmacokinetic properties using various computational techniques.

## 2. Materials and Methods

### 2.1. Protein Preparation

For the current study, the CryoEM structure of the human SLC4A4 sodium-coupled acid-base transporter NBCe1, represented by PDB ID 6CAA, was selected, on the basis of multiple colorectal gene expression data from the Gene Expression Omnibus (GEOs) repository [35]. However, to optimize the 3D protein structure of 6CAA and assess the presence of any gaps or missing residues, homology modeling was conducted using the SWISS-MODEL [36].

To find evolutionarily comparable structures that fit with the target sequence, the SWISS-MODEL (SMTL version 2024-03-27, PDB release 22 March 2024) template library was used in this approach. A total of 279 templates were initially identified, from which the top template was filtered using a heuristic approach.

### 2.2. Receptor-Based Screening

In our receptor-based screening process, we capitalized on the 3D structures of the predicted receptor, obtained from the SWISS-MODEL of the 6CAA protein, to pinpoint potential binding pockets. Subsequently, we executed high-throughput docking experiments, employing the experimental drug library sourced from the DrugBank database (version 5.1.7) [37]. These docking experiments were facilitated by the DrugRep software, which harnesses CurPocket for pocket detection and Autodock Vina for molecular docking [38].

### 2.3. Ligand Preparation from Experimental Drug Library

In our investigation, the preparation of ligands from the experimental drug library retrieved from the DrugBank database (version 5.1.7) served as a pivotal stage in performing high-throughput docking experiments utilizing the DrugRep software with default settings. The initial hit, often indicative of the ligand with the highest docking score, signifies a robust predicted binding affinity to the protein target. Elevated scores imply a more advantageous interaction between the ligand and the protein’s binding site. Consequently, we selected the first hit for subsequent exploration as a potential drug candidate.

### 2.4. Molecular Docking with CB-DOCK2

CB-DOCK2 was used to molecular dock the first hit, DB07991, which was found by DrugRep, in order to obtain the drug–ligand complex. Autodock Vina, based this step, aimed to refine the binding conformation of the ligand within the protein’s binding site, providing insights into the stability and interactions of the drug–ligand complex [39].

### 2.5. Molecular Dynamics Simulation

The MD simulations utilized GROMACS version [2020.1-Ubuntu-2020.1-1 and 2024.1-dev] (www.gromacs.org accessed on 19 march 2023), with the charmm36-jul2022.ff force field, to analyze the MD simulation [40]. The starting structure of the protein–ligand complex was obtained from the CB-DOCK2 software. The protein and ligand structures were prepared using the pdb2gmx tool, and missing hydrogen atoms were added. A single 50-nanosecond MD simulation was conducted to evaluate the dynamic behavior of the complex over time. The parameter files and topology of ligands were prepared utilizing the latest CgenFF via CHARMM-GUI [41]. The complex was solvated in a cubic box with the appropriate water model (e.g., TIP3P), ensuring a minimum of 10 Å between the protein and the box edge. Sodium ions were added to neutralize the system’s charge.

To introduce periodic boundary conditions to both systems, a Leapfrog MD integrator was used for 50 ns during the NPT/NVT equilibration run. The particle-mesh Ewald technique was employed to account for electrostatic forces. The non-bonded contacts above 10 and 12 Å were terminated using the force-based switching technique. To remove faulty connections from the system, the steepest drop, with 50,000 steps, was used to minimize energy consumption. The gmxrms and gmxrmsf tools were utilized to compute the receptor’s root mean square deviation (RMSD) and root mean square fluctuation (RMSF), respectively. The gmxhbond tool was used to examine hydrogen bonding. The radius of gyration (Rg) and solvent-accessible surface area (SASA) were calculated, respectively, using the gmxsasa and gmxgyrate programs. With Grace Software (Grace-5.1.25), plots were created [42]. The complex structure was visualized using PyMol [43]. The simulation time for the ligand–receptor interaction was 22 h, for a duration of 50 nanoseconds, over the GridMarkets server.

## 3. Results

### 3.1. Molecular Modeling

**Figure 1:**
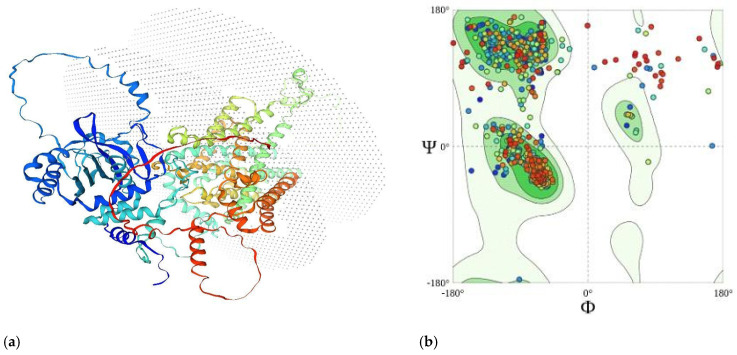
(**a**) Q9Y6R1.1.A template-based model of SLC4A4 protein (6CAA); (**b**) Ramachandran Favoured plot for model, based on Q9Y6R1.1.A template.

SLC4A4 underwent 3D modeling with reference to the template i.e., Q9Y6R1; its specifications are mentioned in Table 1. The structure was assessed using the Protein Structure and Model Assessment tools of the SWISS-MODEL Server, which showed good scores, such as a Global Model Quality Estimate of 0.71 and a Ramachandran Favoured of 90.69%. Also, the MolProbity Score was 1.64 (Table 2, Figure 1a,b). These evaluations highlighted the applicability of the model for further structure-based 6CAA analysis and optimization.

### 3.2. Molecular Docking

A cavity-detection guided receptor-based screening identified the docking pockets of the protein receptor using CB-Dock, as detailed in Table 3. It screened for potential leads by examining the interaction of pocket 1. DB07991 achieved the highest docking score of −10 against the modeled 6CAA when docking was performed using DrugRep, which utilized AutoDock Vina (version 1.1.2) as its backend. This high score is attributed to the strong hydrogen bonding and hydrophobic interactions observed at pocket 1. This screening was conducted against an experimental drug library, containing 5935 drugs, from the DrugBank database (version 5.1.7), as shown in Table 4.

**Figure 2:**
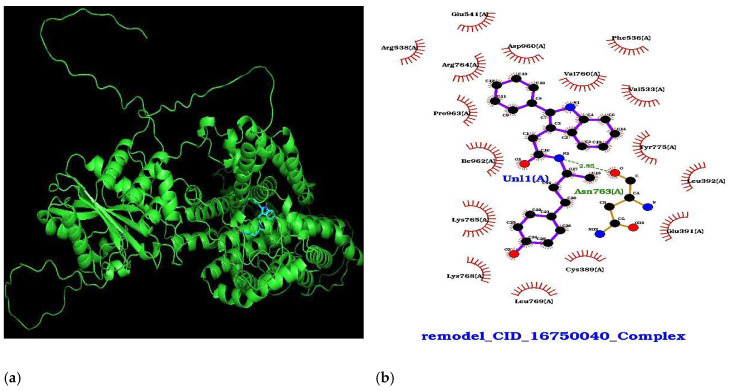
(**a**) Complex of modeled 6CAA and DB07991—visualization through Pymol; (**b**) molecular lever interaction of 6CAA protein and DB07991—visualization through LigPlot+.

To obtain the best docking pose, redocking with DB07991 was performed with CB DOCK2, and the protein–ligand complex was obtained, as depicted in Figure 2a,b, with the help of PyMOL v3.0 and ligplot+ version 2.2.8 [44,45]. The docked complex of the modeled 6CAA and DB07991 was further studied using molecular dynamic simulation.

### 3.3. Molecular Dynamic Simulation

**Figure 3:**
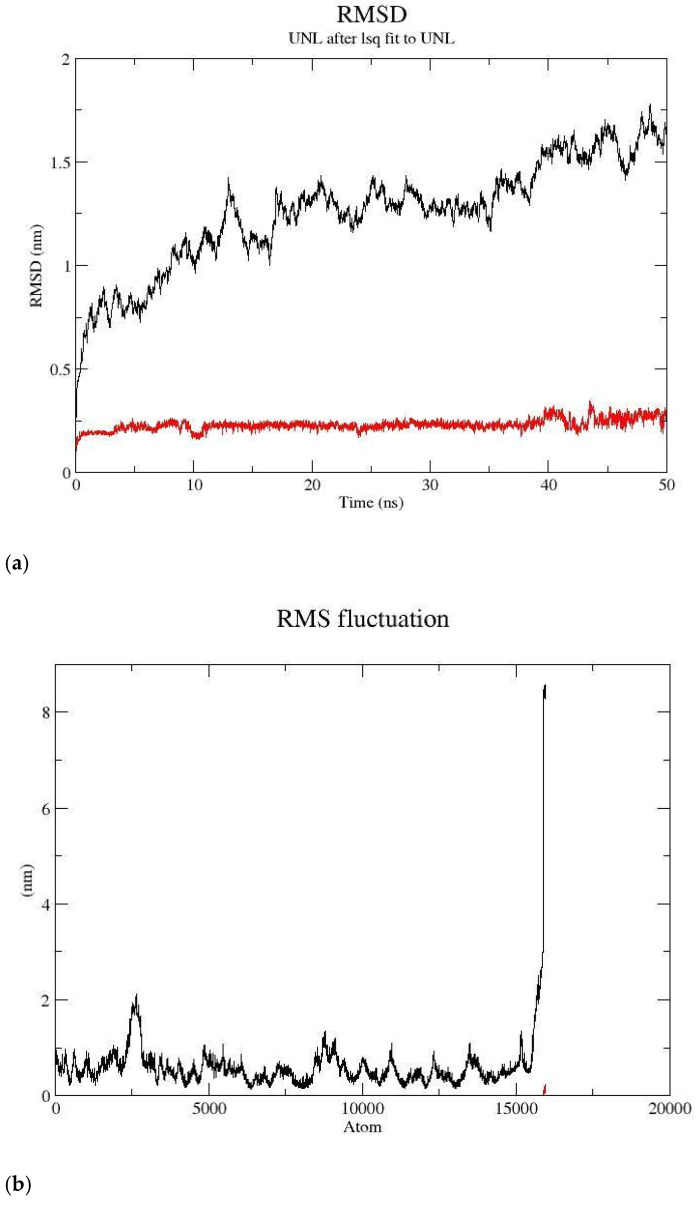

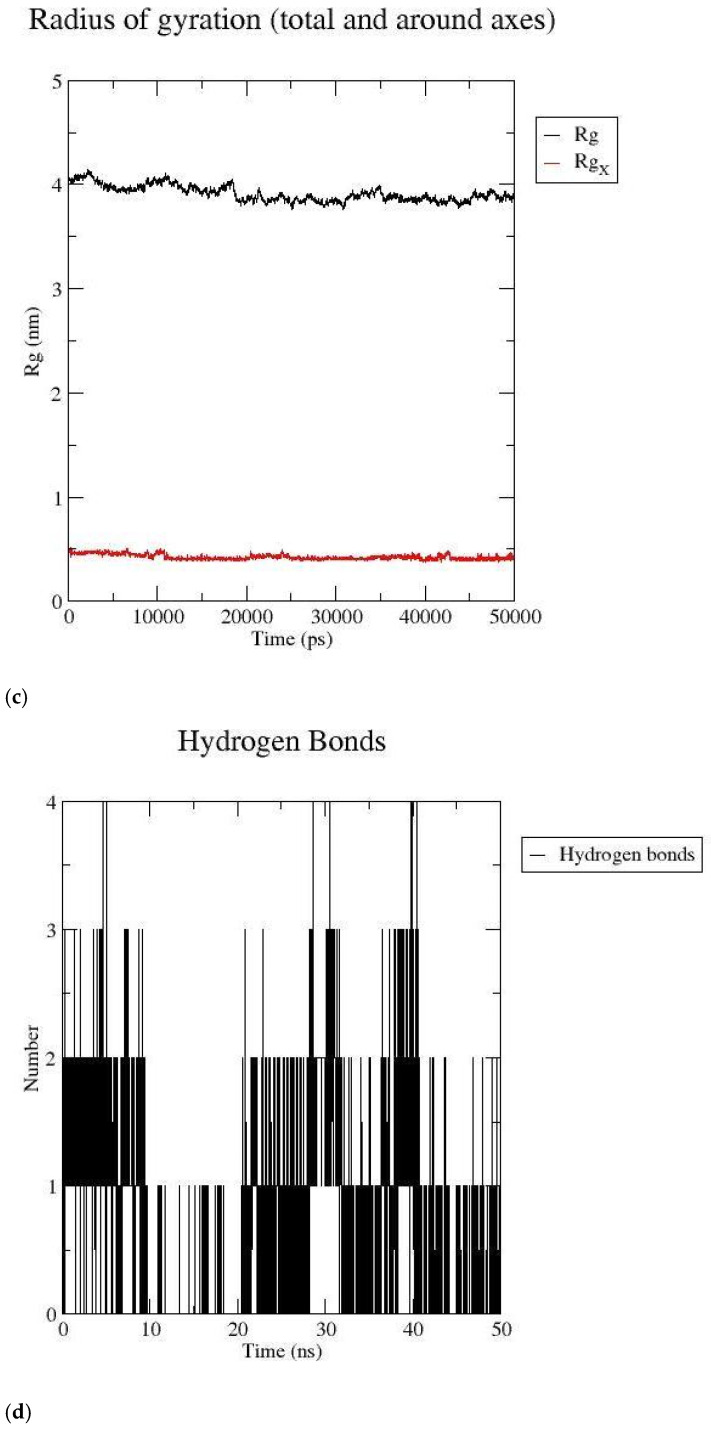

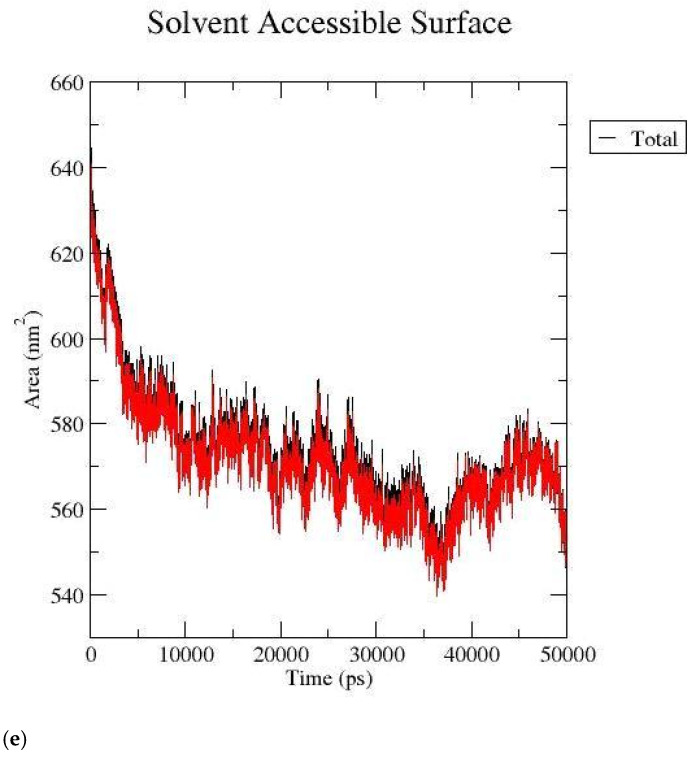
(**a**) Root mean square deviation of modeled 6CAA protein and ligand DB07991. (**b**) Root mean square fluctuation of atoms during MD simulation process of modeled 6CAA protein and ligand DB07991. (**c**) Radius of gyration (Rg) plots for modeled 6CAA protein and ligand DB07991. (**d**) Hydrogen bond plots for modeled 6CAA protein and ligand DB07991. (**e**) Solvent-accessible surface area for modeled 6CAA protein and ligand DB07991.

Over a period of time, studies based on molecular dynamics provided a profound understanding of the interactions between proteins and ligands. Here, in the case of protein, i.e., modeled 6CAA and ligand DB07991, the complex exhibited an acceptable range of deviation, with the protein experiencing a maximum deviation of ~1.5 nm RMSD throughout the 50 ns black-color graph, and the ligand kept iterating within ~0.25 nm RMSD during the entire period of the 50 ns red-color graph shown in Figure 3a. The overall RMSD depicts the ligand movement within the binding site of the protein.

The mean variations in atoms or amino acid residues over the course of the MD simulation were computed using the root mean square fluctuation (RMSF). Here, the protein N-terminal showed minor fluctuations of 1.0 nm along the entire length of the protein, and the C-terminal exhibited up to ~8.0 nm of fluctuations on the black-color graph, whereas the ligand exhibited under ~0.5 nm of fluctuations on the red-color graph in Figure 3b.

The Rg (radius of gyration) can define the compactness of the protein and bound ligand, and is helpful for understanding the unfolding and folding of the protein–ligand complex. Here, the ligand exhibited a movement of up to 0.5 nm, and the protein experienced a movement of 4.0 to 3.9 nm at the last conformation; the ligand’s movement within the protein’s binding site region is depicted in Figure 3c.

Bonding parameters are a crucial part of drug discovery, as they help to provide a better understanding of interaction, absorption, and multiple processes. Here, the ligand experienced a maximum of 04 h-bonds at the nearly 5 ns, 28 ns, 30 ns, 39 ns, and 42 ns periods. Finally, at the last conformation at 50 ns, it experienced 02 h-bonds, as depicted within the protein–ligand complex in Figure 3d.

Regarding solvent or water accessibility, the change in conformation of the protein and ligand conformation can be detected and assessed by SASA (solvent-accessible surface area). In the current study, the protein in the solvent system is shown by the black line graph, and the protein and ligand complex is shown by the red line graph, for the duration of the MD simulation. The overlap demonstrates the system’s overall stability and the absence of any or presence of only very little oscillations Figure 3e.

**Figure 4:**
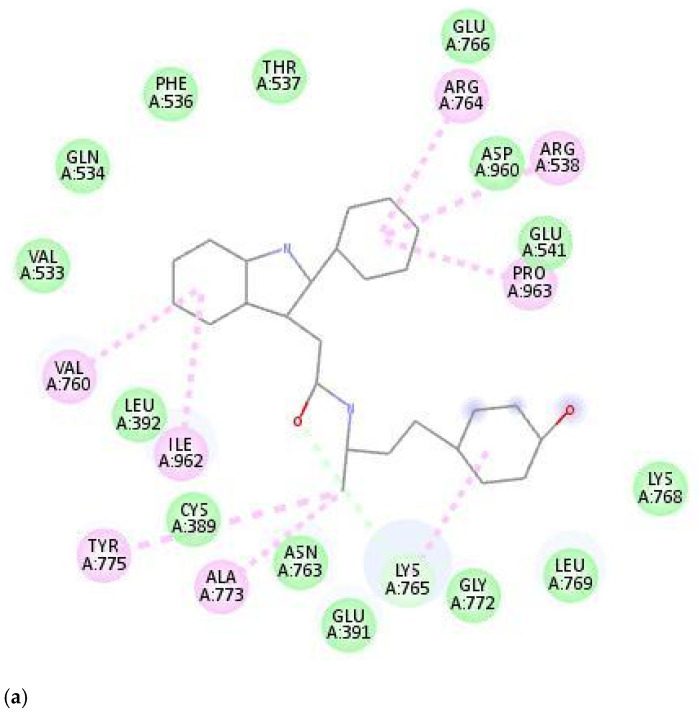

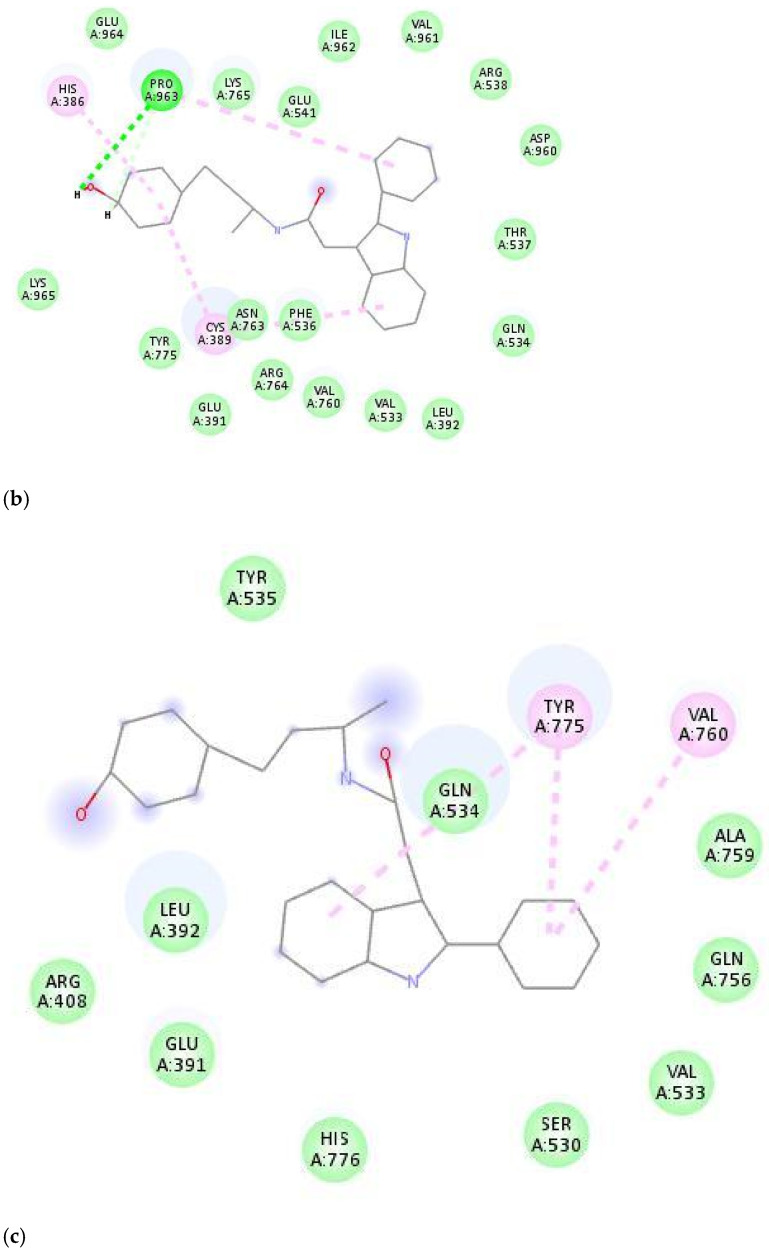
(**a**) Conformation at 0th ns. (**b**) Conformation at 25th ns. (**c**) Conformation at 50th ns.

Initially, after the energy minimization in the complex, the compound exhibited hydrogen bonding with Lys765, pi-interactions with Arg538, Val760, Arg764, Lys765, Ala773, Tyr775, Ile962, and Pro963, and Vanderwaals interactions with Glu391, Leu392, Val533, Gln534, Phe536, Glu541, Thr537, Glu766, Asn763, Lys768, Leu769, Gly772, and Asp960 (Figure 4a).

At 25 ns, the compound exhibited two hydrogen bonds with Pro963, pi-interactions with His386, Cys389, and Pro963, and Vanderwaal interactions with Glu391, Leu392, Val533, Gln534, Phe536, Thr537, Arg538, Glu541, Asn763, Lys765, Tyr775, Asp960, Val961, Ile962, Glu964, and Lys965 (Figure 4b).

At the final and 50th ns, the compound exhibited no hydrogen bonding within the binding site region, pi-interactions with Val 760 and Tyr775, and Vanderwaal interactions with Glu391, Leu392, Arg408, Ser530, Val533, Gln534, Tyr535, Gln756, Ala759, and His776 (Figure 4c).

From the obtained conformations at the 0th ns, 25th ns, and final 50th ns, the compound was observed to be oscillating within the binding site region of the protein. The above figures depict the compound residing with the binding site region of the protein and exhibiting all of its plausible interactions.

## 4. Discussion

Drug repositioning, or repurposing, is an efficient strategy for identifying new therapeutic uses for existing approved or experimental drugs, significantly accelerating the drug discovery process. Targeting SLC4A4 in colorectal cancer (CRC) therapy could play a crucial role in regulating tumor acidity and modulating immune responses within the tumor microenvironment (TME). As a bicarbonate transporter, SLC4A4 contributes to maintaining pH balance, which is vital for cancer cell survival, and impacts the acidic environment that typically suppresses immune cell activity and promotes immune evasion. Furthermore, its association with the infiltration of immune cells, such as CD8+ T cells, dendritic cells, macrophages, NK cells, and Th1 cells, suggests that SLC4A4 influences the inflammatory characteristics of the TME. This protein has also been linked to enhanced responses to immune checkpoint inhibitors like nivolumab and ipilimumab, highlighting its potential to improve immunotherapeutic outcomes. By modulating both pH dynamics and immune cell activity, SLC4A4 emerges as a promising therapeutic target for CRC; however, experimental studies are needed to validate its physiological impact and therapeutic efficacy further [46].

The molecule DB07991, although its pharmacological actions are not well understood, has been reported in the DrugBank database to target the estrogen receptor ESR1 [47]. Together, molecular docking and molecular dynamics simulations serve as indispensable computational tools in modern drug discovery and repurposing efforts. These computational methods provide detailed insights into the interaction patterns of potential drug candidates with their target proteins. In this study, molecular docking and dynamics simulations were employed to investigate the interaction between DB07991 and the modeled SLC4A4 protein (6CAA) [35]. The results from these computational studies offer a comprehensive understanding of how DB07991 interacts with the SLC4A4 protein, revealing stable binding and consistent hydrogen bonding patterns. These findings suggest that DB07991 has the potential to inhibit SLC4A4 function effectively, making it a promising candidate for further exploration as an anticancer drug.

While this study provides valuable insights into the potential of DB07991 as a therapeutic candidate for CRC, the findings are limited by the in silico nature of the analysis. Computational docking and molecular simulations, while informative, may not fully capture the complexity of biological interactions. Experimental validation is essential to confirm the efficacy and safety of DB07991.

Future studies should focus on validating the efficacy of DB07991 using advanced experimental models, such as organs-on-chips, cell lines, and colorectal cancer-induced animal models. These systems will provide a more comprehensive understanding of the drug’s toxicity, pharmacokinetics, and efficacy, paving the way for preclinical studies.

## 5. Conclusions

In this study, molecular docking and dynamics simulations were employed to investigate the interaction of DB07991 with the SLC4A4 protein (6CAA). The results indicate that DB07991 exhibits high binding affinity and stability, maintaining a consistent binding pose throughout the simulations. Notably, the molecule forms strong hydrogen bonds with the target protein, contributing to its stable interaction. The protein’s flexibility does not negatively impact ligand binding, suggesting that DB07991 can effectively interact with SLC4A4 without causing significant conformational distortions. Based on these findings, DB07991 is identified as a promising drug candidate for further exploration as a potential therapeutic agent against colorectal cancer. However, experimental validation is required to confirm its pharmacological efficacy and safety in biological systems.

## Figures and Tables

**Table 1:** Template specification with respect to SLC4A4 gene (6CAA).

Template	Seq Identity	Oligo-State	Found by	Method	Resolution	Seq Similarity	Range	Coverage	Description
Q9Y6R1.1.A	99.5	Monomer	AFDB search	AlphaFold v2	-	0.61	35–1035	0.97	Electrogenic sodium bicarbonate cotransporter 1

**Table 2:** Built model 1 information.

Model	Built with	Oligo-State	Ligands	GMQE	MolProbity Score	Ramachandran Favoured
1	ProMod3 3.4.0	Monomer	None	0.71	1.64	90.69%

**Table 3:** Predicted binding pockets.

Pocket	Volume	Center	Size
1 (Chain A (G385 H386 G387 D388 C389 E390 E391 L392 Q393 F531 L532 V533 Q534 Y535 F536 T537 R538 F539 T540 E541 I757 A759 V760 N763 R764 K765 Y775 R881 F909 R943 K944 D947 Q952 L955 S956 F957 L958 D959 D960 V961 I962 P963 E964 K965 D966 K967 K970))	3676	−1.4, −18.4, −0.4	22, 21, 19
2 (Chain A (LYS103 ILE217 THR172 PRO820 LYS233 SER218 GLY268 LYS173 SER133 SER134 ARG86 LEU270 ASP216 ASP219 LEU135 HSD170 GLU92 PRO104 ASN228 ALA269 LYS229 LYS227 PRO221 LYS220 LYS167 ARG166 GLU272 LEU165 SER102 TRP101 LEU267 ARG169 PHE230 ASP224 PRO136 HSD168 GLN225 GLU222 MET266))	3544	−38.7, 12.7, 19.0	30, 16, 16
3 (Chain A (MET813 LEU827 PHE431 ILE761 SER545 SER427 LYS812 LYS681 ASP809 THR677 LYS667 ASP685 ARG680 ARG830 PHE826 GLU814 SER810 ALA871 VAL876 MET868 HSD767 SER872 GLU766 ARG764 GLY875 GLU541 LYS670 GLU542 TYR867 ASN874 GLN424 SER684 ILE688 ILE808 VAL829 ILE805 ALA806))	2519	−8.8, 4.3, −0.6	24, 19, 18
4 (Chain A (THR302 LYS93 ILE334 HSD105 PHE90 TRP87 VAL94 ILE88 GLU95 ASP311 GLN63 SER110 GLU295 ARG298 LEU114 THR108 VAL106 LEU111 LEU336 PRO343 ILE345 PHE329 LEU330 LYS360 TRP341 LYS89 ALA344 ILE347 PHE115 GLY339 LEU64 PRO62 ASP342 HSD310 ARG346 GLU91 SER102 VAL333 GLU307 LEU109 VAL335 SER305 ASP306 MET304 PRO337 PRO61 ALA107 PRO338))	2046	−23.4, 1.9, 27.0	19, 27, 25
5 (Chain A (VAL927 ILE551 GLY486 VAL802 SER484 PRO487 LEU489 LEU446 PHE552 ASP555 PHE443 ASN439 ILE548 LEU494 LYS558 LYS559 ASN497 SER925 THR926 LYS924 ALA450 THR801 ASP449 TYR554 VAL490 ALA929 TRP921 ARG493 ALA800 THR485))	1503	14.7, 5.1, −14.3	20, 18, 15

**Table 4:** Top 10 hits by DrugRep for pocket 1.

Drugbank ID	Name	Formula	Dock Score	MW	LogP
DB07991	N-[(1R)-3-(4-HYDROXYPHENYL)-1-METHYLPROPYL]-2-(2-PHENYL-1H-INDOL-3-YL)ACETAMIDE	C26H26N2O2	−10	398.5	5.3
DB07213	(5-{3-[5-(PIPERIDIN-1-YLMETHYL)-1H-INDOL-2-YL]-1H-INDAZOL-6-YL}-2H-1,2,3-TRIAZOL-4-YL)METHANOL	C24H25N7O	−9.9	427.5	2.5
DB06925	3-(2-AMINOQUINAZOLIN-6-YL)-4-METHYL-N-[3-(TRIFLUOROMETHYL)PHENYL]BENZAMIDE	C23H17F3N4O	−9.8	422.4	4.9
DB02112	Zk-806450	C31H31N5O	−9.7	489.6	5.2
DB14070	HM-30181	C38H36N6O7	−9.6	688.7	4.7
DB02555	SP4160	C33H42Cl2N8O4	−9.6	685.6	3.5
DB07145	(2R)-N-HYDROXY-2-[(3S)-3-METHYL-3-{4-[(2-METHYLQUINOLIN-4-YL)METHOXY]PHENYL}-2-OXOPYRROLIDIN-1-YL]PROPANAMIDE	C25H27N3O4	−9.5	433.5	3.2
DB02169	9,10-Deepithio-9,10-Didehydroacanthifolicin	C44H68O13	−9.4	805	3.4
DB02729	SD146	C49H44N8O5	−9.4	824.9	6.7

## Data Availability

All data are available from the authors and shall be provided upon request.
